# Early characterisation and prediction of liver diseases in pregnancy by plasma cell‐free RNAs

**DOI:** 10.1002/ctm2.1439

**Published:** 2023-10-13

**Authors:** Jinghua Sun, Wen‐Jing Wang, Xuanyou Zhou, Tingyu Yang, Mujin Ye, Weihui Shi, Zhongzhen Liu, Lanlan Zhang, Qing Zhou, Yulong Qin, Yiyao Chen, Fang Chen, Hefeng Huang, Songchang Chen, Chenming Xu

**Affiliations:** ^1^ BGI‐Shenzhen Shenzhen Guangdong P. R. China; ^2^ Obstetrics and Gynecology Hospital Institute of Reproduction and Development Fudan University Shanghai Shanghai P. R. China; ^3^ College of Life Sciences University of Chinese Academy of Sciences Beijing Beijing P. R. China; ^4^ International Peace Maternity and Child Health Hospital School of Medicine Shanghai Jiao Tong University Shanghai Shanghai P. R. China; ^5^ Research Units of Embryo Original Diseases, Chinese Academy of Medical Sciences (No. 2019RU056) Shanghai China

Dear Editor,

Liver dysfunction during pregnancy can result in adverse pregnancy outcomes. In particular, intrahepatic cholestasis of pregnancy (ICP) increases the risk of stillbirth, while hepatitis B virus (HBV) infection raises the likelihood of developing ICP.^1^ Despite extensive research, the molecular pathways underlying ICP are not fully understood, and early predictive biomarkers for ICP are still unavailable. Here, we conducted a comparative analysis of plasma cell‐free RNA (cfRNA) from ICP (N = 74) or HBV (N = 40) patients and healthy pregnant women (N = 171) to explore the biological processes involved in ICP and HBV infection (Supporting Information Figure S1). We identified cfRNA biomarkers to predict ICP before the onset of symptoms, demonstrating the potential clinical applications of cfRNA in liver diseases.

The detailed methods are described in Supporting Information. Nineteen ICP patients with a total bile acids (TBA) concentration of more than 10 μmol/L (ICP_TBA > 10 group) and 55 patients with normal TBA levels (TBA < 10 μmol/L, pre‐ICP group) at sampling time were included in the ICP group (Figure 1A). The average diagnosis time of patients in the ICP_TBA > 10 group was 10.89 gestational weeks, which was significantly earlier than that of the pre‐ICP group (33.20 gestational weeks) (*p* = 1.05E‐10). The detailed clinical information of ICP patients is summarized in Supporting Information Table S1. Differentially abundant genes (DAGs) analysis identified 34 genes with altered abundance in the plasma of ICP patients (Figure 1B, Supporting Information Figure S2A and Table S2). Some DAGs are involved in transmembrane transport, biosynthesis and metabolism (Figure 1C). Gene set enrichment analysis (GSEA) of mRNA and enrichment analysis of differentially abundant miRNAs (DA‐miRNAs) showed that pathways related to immune response, cell junction, extracellular matrix organization, biosynthesis and metabolism, transmembrane transport and coagulation were enriched among genes found to be more abundant in patients with ICP, which reflects the known pathogenesis of ICP (Figure 1D and Supporting Information Figure S2B).^2^, ^3^ Single‐sample GSEA (ssGSEA) analysis found that most of the pathways related to liver function were only significantly increased in the pre‐ICP group but not in the early‐diagnosed ICP group, consistent with the differences in clinical liver transferases, implying the heterogeneity between these two groups (Figure 1E and Supporting Information Table S1).

**FIGURE 1:**
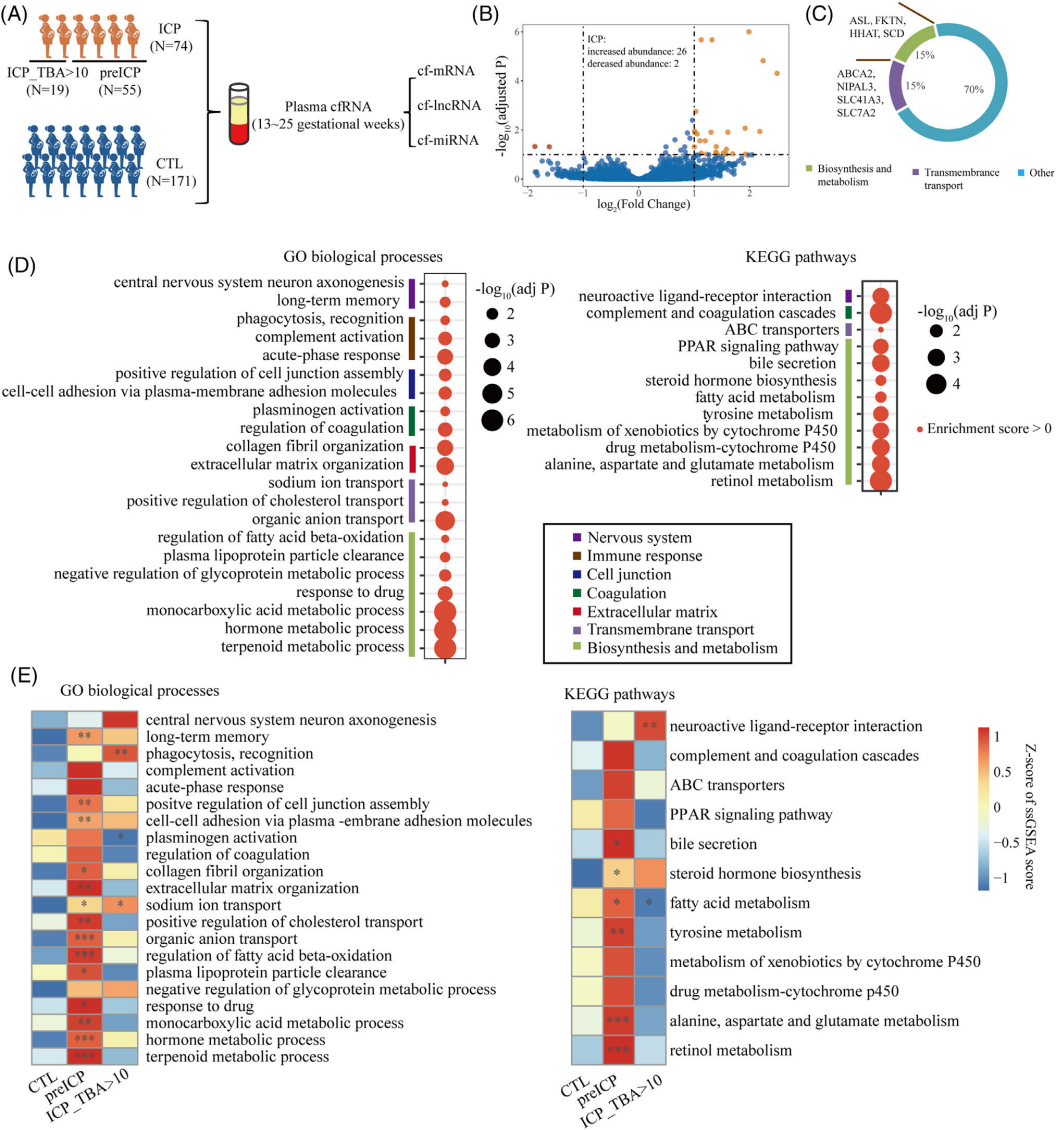
Differentially abundant cf‐mRNAs and functional signaling pathways of ICP. (A) Schematic diagram showing samples and analysis procedure for ICP study. (B) Volcano plot of the differentially abundant cf‐mRNAs and cf‐lncRNAs of ICP. (C) The functional annotation of differentially abundant mRNAs. (D) Pathway enrichment using GSEA. The fold change of genes (ICP/CTL) was calculated using DESeq2. (E) Heatmap showing the mean ssGSEA score for CTL, pre‐ICP and ICP_TBA > 10 groups. The *p*‐value was calculated using Wilcoxon's rank‐sum test. ***Indicates *p* < .001; **p < .01; *p < .05.

Ten samples with positive HBeAg and 30 samples with negative HBeAg were included in the HBV group (Figure 2A). The detailed clinical information of HBV patients is summarized in Supporting Information Table S3. HBV RNA can be detected in 28 (70%) HBV samples and the HBeAg‐positive samples presented a higher abundance than the HBeAg‐negative samples (Figure 2B). Seven samples were assembled successfully and determined the HBV genotype (Supporting Information Figure S3A). Their sequencing coverage was uniform, avoiding technical errors in sequencing (Supporting Information Figure S3B & C). DAG analysis revealed 125 genes with altered abundance in HBV patients including well‐established liver damage biomarkers (Figure 2C, Supporting Information Figure S4A and Table S2).^4^ The DAGs annotation and GSEA showed that molecular changes of HBV were associated with liver function and cell apoptosis, and these pathways were more significant in HBeAg‐positive samples (Figure 2D‐F). The enrichment pathway of DA‐miRNA target genes is similar to that of the mRNA GSEA result (Supporting Information Figure S4B‐C). Among the DAGs, *DCTN3*, *PROESR3*, *SCD*, *SLC7A2* and *hsa‐miR‐4443* also increased their abundance in the ICP patients, suggesting a shared molecular alteration in the pathogenesis of HBV and ICP (Supporting Information Table S2). Meanwhile, pathways related to biosynthesis and metabolism, transmembrane transport and complement and coagulation cascades were enriched in both HBV and pre‐ICP groups, indicating a shared mechanism of HBV and ICP.

**FIGURE 2:**
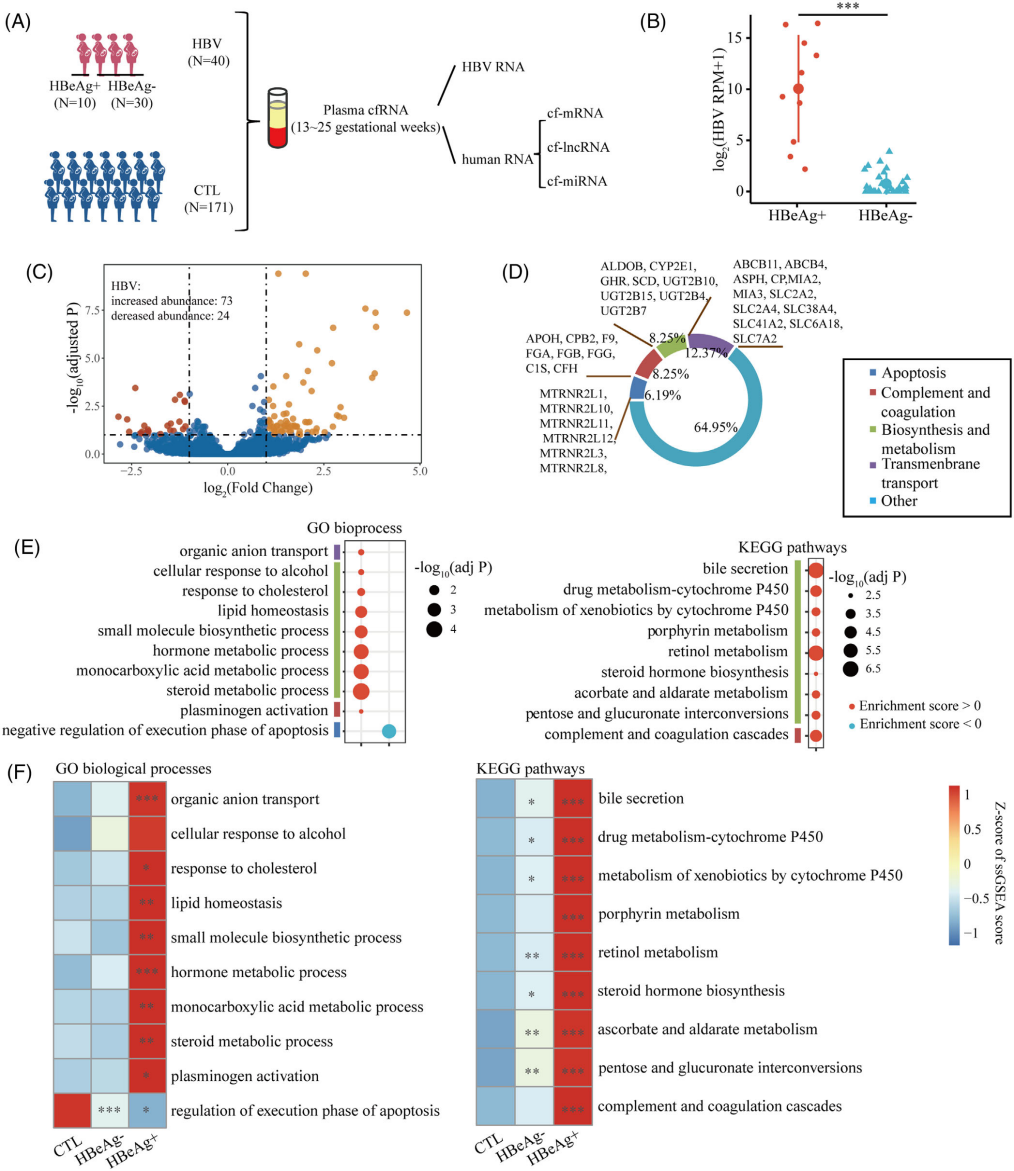
Differentially abundant cf‐mRNAs and cf‐lncRNA and functional signaling pathways of HBV. (A) Schematic diagram showing samples and analysis procedure for HBV study. (B) The box plots of HBV RNA levels in patients with positive or negative HBeAg. The *p*‐value was calculated using Wilcoxon's rank‐sum test. ***Indicates *p* < .001. (C) Volcano plot of the differentially abundant cf‐mRNAs and cf‐lncRNAs. (D) The functional annotation of differentially abundant mRNAs. (E) Pathway enrichment using GSEA. The genes fold change (HBV/CTL) was calculated using DESeq2. (F) Heatmap showing the mean ssGSEA score for CTL, HBV with negative HBeAg and HBV with positive HBeAg groups. The *p*‐value was calculated using Wilcoxon's rank‐sum test. ***Indicates *p* < .001; **p < .01; *p < .05.

We next investigated the feasibility of using cfRNA as a non‐invasive tool for assessing liver health. Only one DAG in ICP was liver‐specific, while 18 (21%) DA‐mRNAs and 5 (18%) DA‐miRNAs were liver‐specific in HBV, and these genes were upregulated in both HBV and ICP (Figure 3A‐C). Additionally, the liver and hepatocyte‐specific signature increases in the HBV and pre‐ICP groups and the hepatocyte score were positively correlated with alanine aminotransferase, triglyceride, γ‐glutamyl transpeptidase and HBV RNA level (Figure 3D‐F and Supporting Information Figure S5). Elevated hepatocyte scores from samples with liver cancer further confirmed that hepatocyte signatures are a robust indicator to assess liver health status (Figure 4G).^5^, ^6^ The placental and fetal‐specific signatures were also investigated. However, no significant alteration was observed except for the fetal liver lymphoid cell (Supporting Information Figure S6), possibly due to the scarcity of fetal signals in early pregnancy and the low TBA concentration.

**FIGURE 3:**
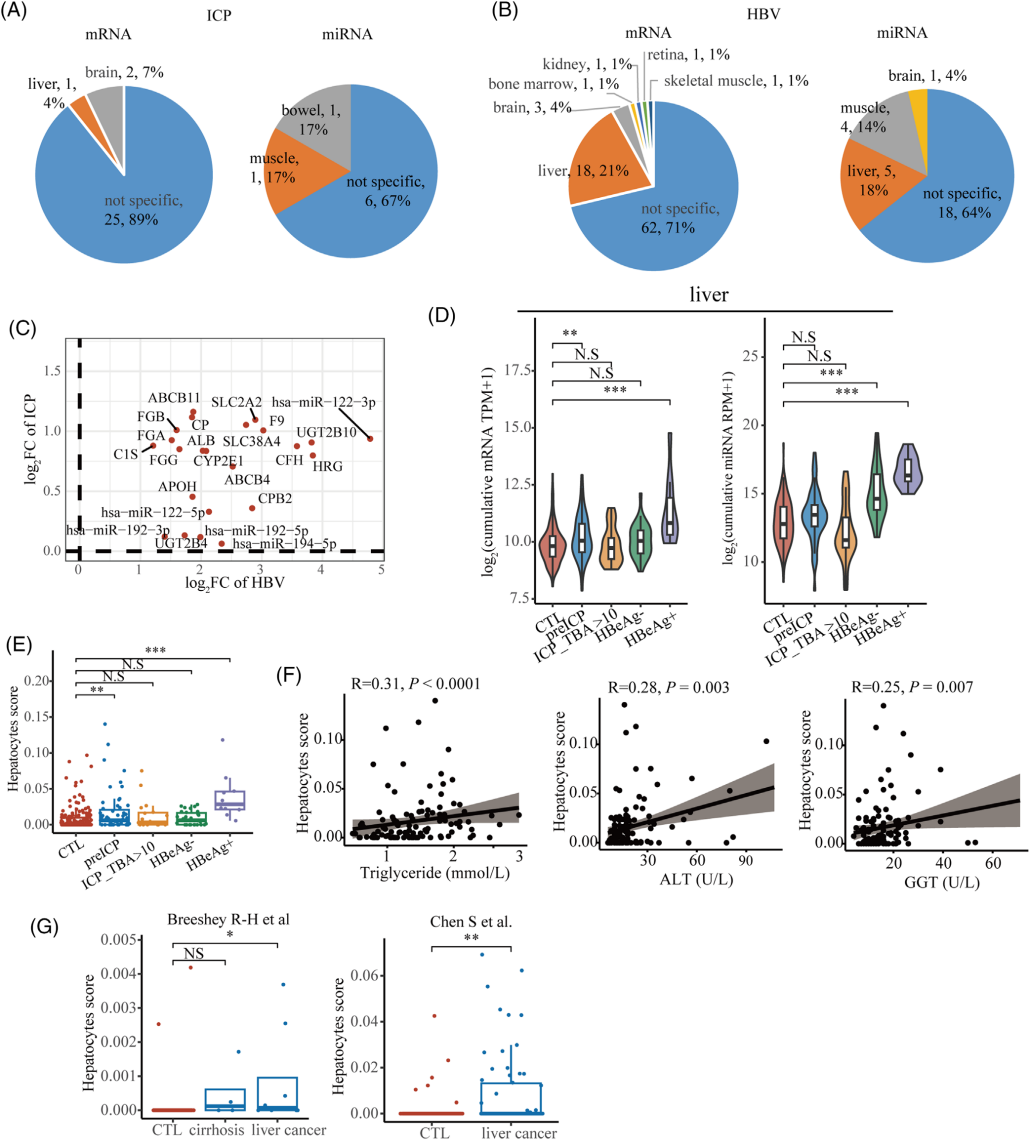
Changes of liver‐specific signature in HBV and ICP patients. (A) Tissue‐specific genes annotation of differentially abundant mRNAs and miRNAs in the ICP group. (B) Tissue‐specific genes annotation of differentially abundant mRNAs and miRNA in HBV group. (C) The fold change of liver‐specific genes in the HBV and ICP group. (D) The cumulative abundance of liver‐specific mRNA and miRNA genes was significantly increased in the HBV and ICP groups. (E) The hepatocyte signature score estimated by xCell was significantly increased in the HBV and ICP groups. (F) The Spearman correlations of hepatocyte score and the clinical liver function indicators. (G) The hepatocyte signature score estimated by xCell was significantly increased in the samples with liver cancer. The *p*‐value was calculated using Wilcoxon's rank‐sum test. *** Indicates *p* < .001; **p < .01; *p < .05; N.S., not significant.

**FIGURE 4:**
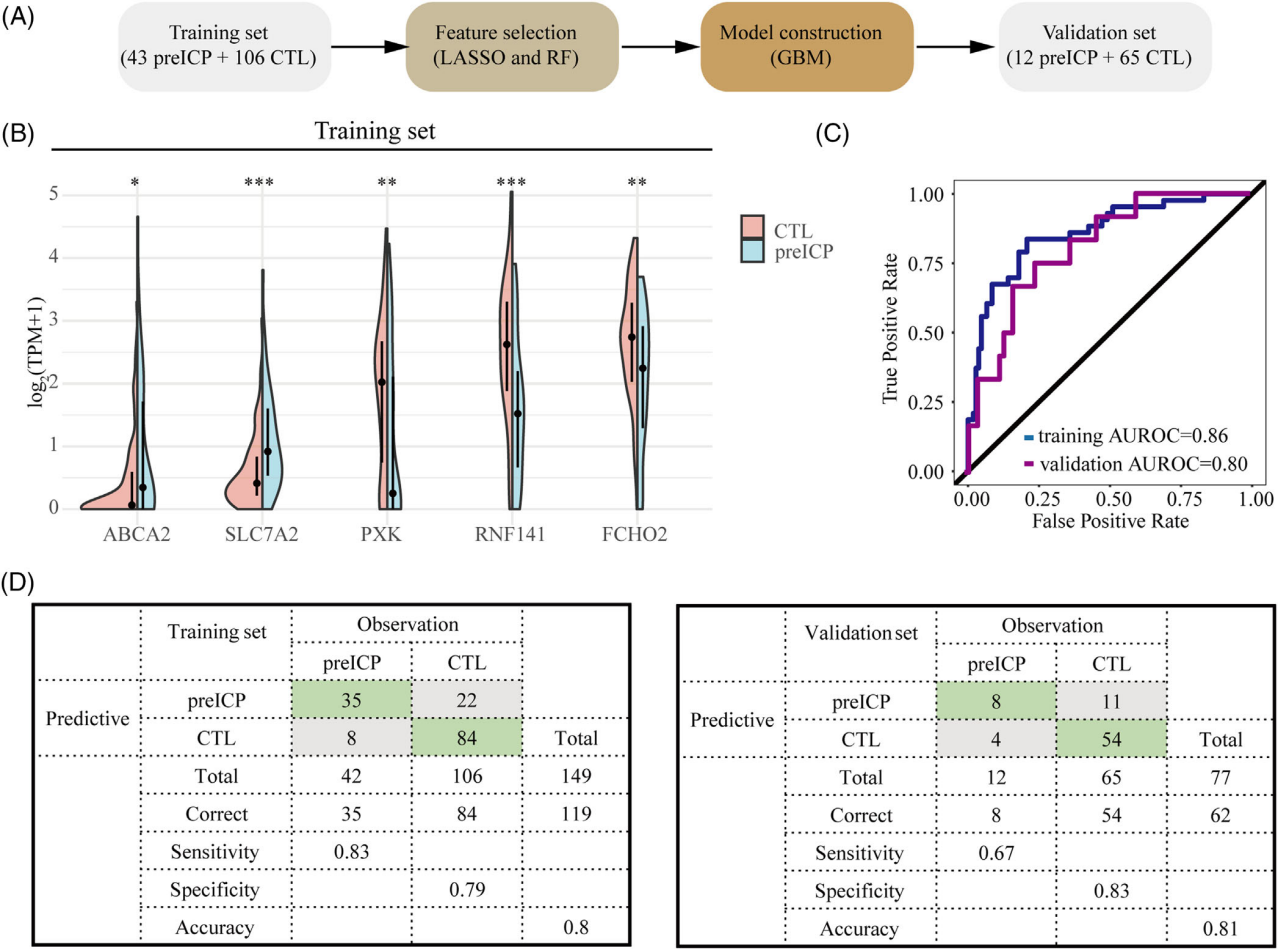
A machine learning model using cf‐mRNA to identify subjects at risk of ICP in early pregnancy. (A) Workflow for building the predictive model. LASSO, least absolute shrinkage and selection operator; RF, random forest; GBM, gradient boosting machine. (B) The box plots of the abundance of mRNA feature genes in the training set. The *p*‐value was calculated using Wilcoxon's rank‐sum test. *** Indicates *p* < .001, **p < .01, *p < .05. (C) The AUROC of the model using cf‐mRNA features. (D) The confusion matrix of the predictive model in the training and validation set. The hyperparameters of the final prediction model using cell‐free mRNA are n. trees = 200, interaction.depth = 1, shrinkage = 0.01 and n.minobsinnode = 10.

To assess whether plasma cfRNA signatures could identify mothers at risk of ICP, we built a machine learning model using samples from the pre‐ICP group and healthy pregnant women. Samples were split into training and validation sets according to the time of blood collection (Figure 4A). A multi‐split method with the least absolute shrinkage and selection operator and random forest algorithms was used to select stable features. A gradient boosting machine with seven‐fold cross‐validation was applied to construct predictive models using mRNA, miRNA and lncRNA, respectively (Figure 4A and Supporting Information Figure S7A). Five mRNA genes (*ABCA2*, *SLC7A2*, *PXK*, *RNF141* and *FCHO2*) were finally selected to construct the model (Figure 4B and Supporting Information Figure S7B). *PXK* and *FCHO2* have been reported to be associated with alkaline phosphatase, aspartate aminotransferase and total cholesterol levels from the genome‐wide association studies.^7^, ^8^, ^9^ The predictive model using mRNA obtained an AUROC of 0.80 and an accuracy of 0.81 in the validation set (Figure 4C&D), which is comparable with the model using clinical features.^10^ The model can be applied to ICP risk stratification after being validated by a large multi‐center population, complementing the efforts based on clinical data. Eight miRNAs were selected to construct the model, but the AUROC is 0.54 in the validation set (Supporting Information Figure S7C and Table S4). LncRNA cannot produce stable features to train a model after multi‐split iterations.

In conclusion, we demonstrated that ICP pathophysiology‐related changes were identified from plasma cfRNAs in early pregnancy. These changes were distinct in patients with early‐ and late‐diagnosed ICP, with the latter showing similar changes to HBV carriers, implicating different biological processes that drive the underlying pathophysiology of these subtypes of ICP. Additionally, plasma cfRNAs provided a non‐invasive means to early identify women at risk for ICP during pregnancy, which could help guide the precision management of pregnancy.

## CONFLICT OF INTEREST STATEMENT

The authors declare that they have no competing interest.

## FUNDING INFORMATION

This work was supported by the research grant from the sub‐project of the National Key R&D Program (2022YFC2703702, 2021YFC2701002), National Natural Science Foundation of China (Nos. 81971344, 82171677, 82192864, 82088102 and 81901495), the Shanghai Municipal Commission of Science and Technology Program (22S31901500, 23ZR1408000, 21Y21901002), Shanghai Municipal Commission of Health and family planning (202140110), and CAMS Innovation Fund for Medical Sciences (2019‐I2M‐5‐064), Collaborative Innovation Program of Shanghai Municipal Health Commission (2020CXJQ01), Clinical Research Plan of SHDC (SHDC2020CR1008A), Shanghai Clinical Research Center for Gynecological Diseases (22MC1940200), Shanghai Urogenital System Diseases Research Center (2022ZZ01012) and Shanghai Frontiers Science Research Center of Reproduction and Development.

## Supporting information


**Supplementary Figure 1. Sample screening flow diagram**.

**Supplementary Figure 2. Differentially abundant cf‐miRNAs and functional signaling pathways of ICP**. (A) Volcano plot of the differentially abundant cf‐miRNAs of subjects with ICP. (B) The enrichment pathway of predicted target genes of differentially abundant cf‐miRNAs of subjects with ICP.

**Supplementary Figure 3. The genotype and reads coverage of HBV RNA**. (A) HBV genotypes determined by cfRNA in HBV‐infected patients. (B‐C) The distribution of cfRNA reads mapping to HBV genotype C (B) and genotype B (C) genome.

**Supplementary Figure 4. The target genes of differentially abundant miRNAs of the HBV groups**. (A) Volcano plot of the differentially abundant cf‐miRNAs of patients with HBV infection. (B) The overlap of target genes of differentially abundant miRNAs and differentially abundant mRNAs in the HBV group. (C) The enrichment pathway of predicted target genes of differentially abundant miRNAs of HBV.

**Supplementary Figure 5. The Spearman correlations of hepatocytes and the HBV RNA level**.

Supplementary Figure 6. Fetal cell‐specific signature scores in the ICP and HBV groups. (A‐B) The cumulative signature score of the fetal liver, brain, lung and heart, and placenta in ICP (A) and HBV (B) groups. X‐axis is the false discovery rate (FDR) comparing the fetal signature score between patients and healthy pregnant women. *P* values were calculated using Wilcoxon's rank‐sum test.

Supplementary Figure 7. The feature selection process of the ICP prediction model. (A) Detailed process of ICP prediction model construction. (B‐C) The frequency of the final feature mRNA (B) and miRNA (C) genes when selected by LASSO and RF algorithms, respectively. LASSO, least absolute shrinkage and selection operator; RF, random forest; GBM, gradient boosting machine.

Supporting Information

Supporting Information

## Data Availability

The plasma cfRNA data that support the findings of this study have been deposited into the Genome Sequence Archive for Human Database with accession numbers HRA003387. Plasma cfRNA data from samples with liver cancer and healthy subjects were downloaded from the Gene Expression Omnibus database with accession numbers GSE182824, GSE174302 and GSE142987. The code of cfRNA alignment and quantification is available at https://github.com/wonderful1/PALM‐Seq‐cfRNA.

## References

[1] Ovadia C , Seed PT , Sklavounos A , et al. Association of adverse perinatal outcomes of intrahepatic cholestasis of pregnancy with biochemical markers: results of aggregate and individual patient data meta‐analyses. Lancet. 2019;393(10174):899‐909. doi:10.1016/S0140-6736(18)31877-4 30773280 PMC6396441

[2] Arrese M , Macias RI , Briz O , Perez MJ , Marin JJ . Molecular pathogenesis of intrahepatic cholestasis of pregnancy. Expert Rev Mol Med. 2008;10:e9. doi:10.1017/S1462399408000628 18371245

[3] Xiao J , Li Z , Song Y , et al. Molecular pathogenesis of intrahepatic cholestasis of pregnancy. Can J Gastroenterol Hepatol. 2021;2021:6679322. doi:10.1155/2021/6679322 34195157 PMC8181114

[4] Mann J , Reeves HL , Feldstein AE . Liquid biopsy for liver diseases. Gut. 2018;67(12):2204‐2212. doi:10.1136/gutjnl-2017-315846 30177542

[5] Roskams‐Hieter B , Kim HJ , Anur P , et al. Plasma cell‐free RNA profiling distinguishes cancers from pre‐malignant conditions in solid and hematologic malignancies. NPJ Precis Oncol. 2022;6(1):28. doi:10.1038/s41698-022-00270-y 35468987 PMC9038724

[6] Chen S , Jin Y , Wang S , et al. Cancer type classification using plasma cell‐free RNAs derived from human and microbes. Elife. 2022;11:e75181. doi:10.7554/eLife.75181 35816095 PMC9273212

[7] Chen VL , Du X , Chen Y , et al. Genome‐wide association study of serum liver enzymes implicates diverse metabolic and liver pathology. Nat Commun. 2021;12(1):816. doi:10.1038/s41467-020-20870-1 33547301 PMC7865025

[8] Willer CJ , Schmidt EM , Sengupta S , et al. Discovery and refinement of loci associated with lipid levels. Nat Genet. 2013;45(11):1274‐1283. doi:10.1038/ng.2797 24097068 PMC3838666

[9] Pazoki R , Vujkovic M , Elliott J , et al. Genetic analysis in European ancestry individuals identifies 517 loci associated with liver enzymes. Nat Commun. 2021;12(1):2579. doi:10.1038/s41467-021-22338-2 33972514 PMC8110798

[10] Zhang X , Chen Y , Salerno S , et al. Prediction of intrahepatic cholestasis of pregnancy in the first 20 weeks of pregnancy. J Matern Fetal Neonatal Med. 2021;35:6329–6335. doi:10.1080/14767058.2021.1911996 34210209

