# Supplementary figures and images for: Early characterisation and prediction of liver diseases in pregnancy by plasma cell‐free RNAs
Source: Clin Transl Med. 2023 Oct 13;13(10):e1439. doi: 10.1002/ctm2.1439 (PMC10570770; doi:10.1002/ctm2.1439)

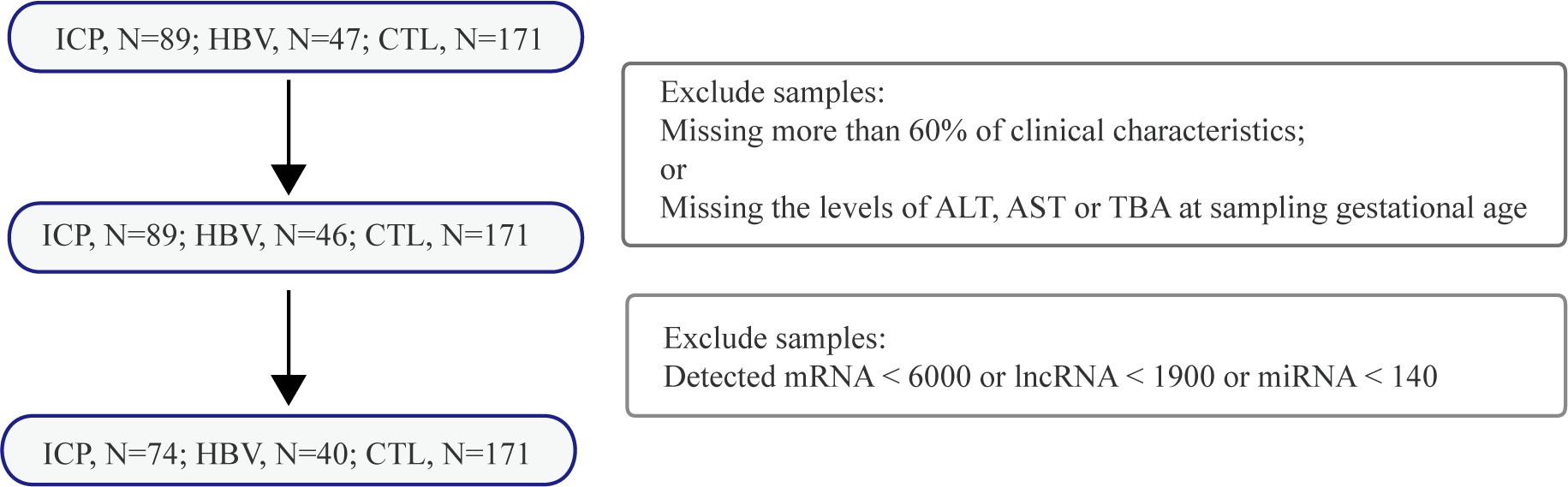

Supplement: Supplementary file 1 — Supplementary Figure 1. Sample screening flow diagram. [file CTM2-13-e1439-s003.tif]

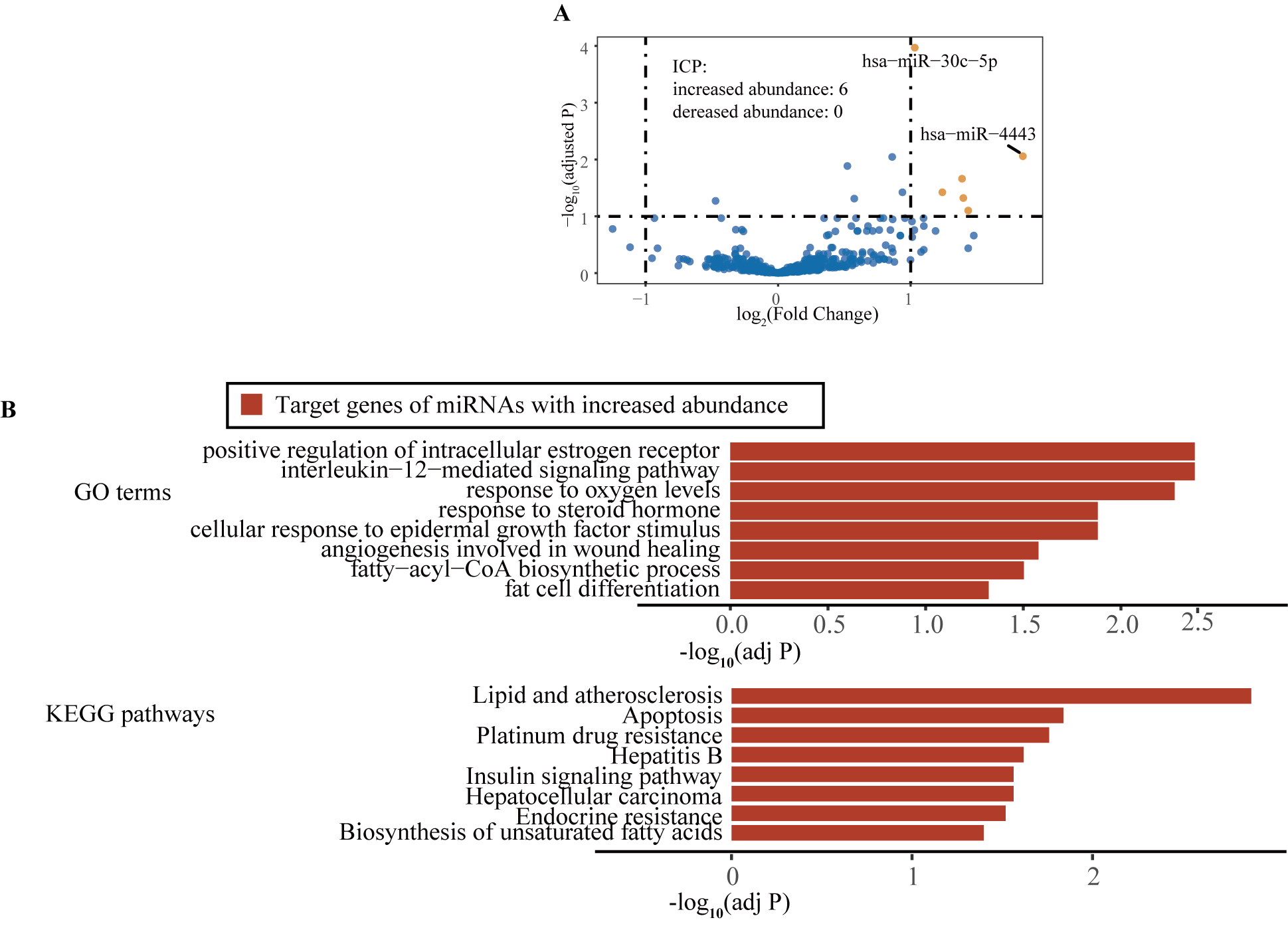

Supplement: Supplementary file 2 — Supplementary Figure 2. Differentially abundant cf‐miRNAs and functional signaling pathways of ICP. (A) Volcano plot of the differentially abundant cf‐miRNAs of subjects with ICP. (B) The enrichment pathway of predicted target genes of differentially abundant cf‐miRNAs of subjects with ICP. [file CTM2-13-e1439-s002.tif]

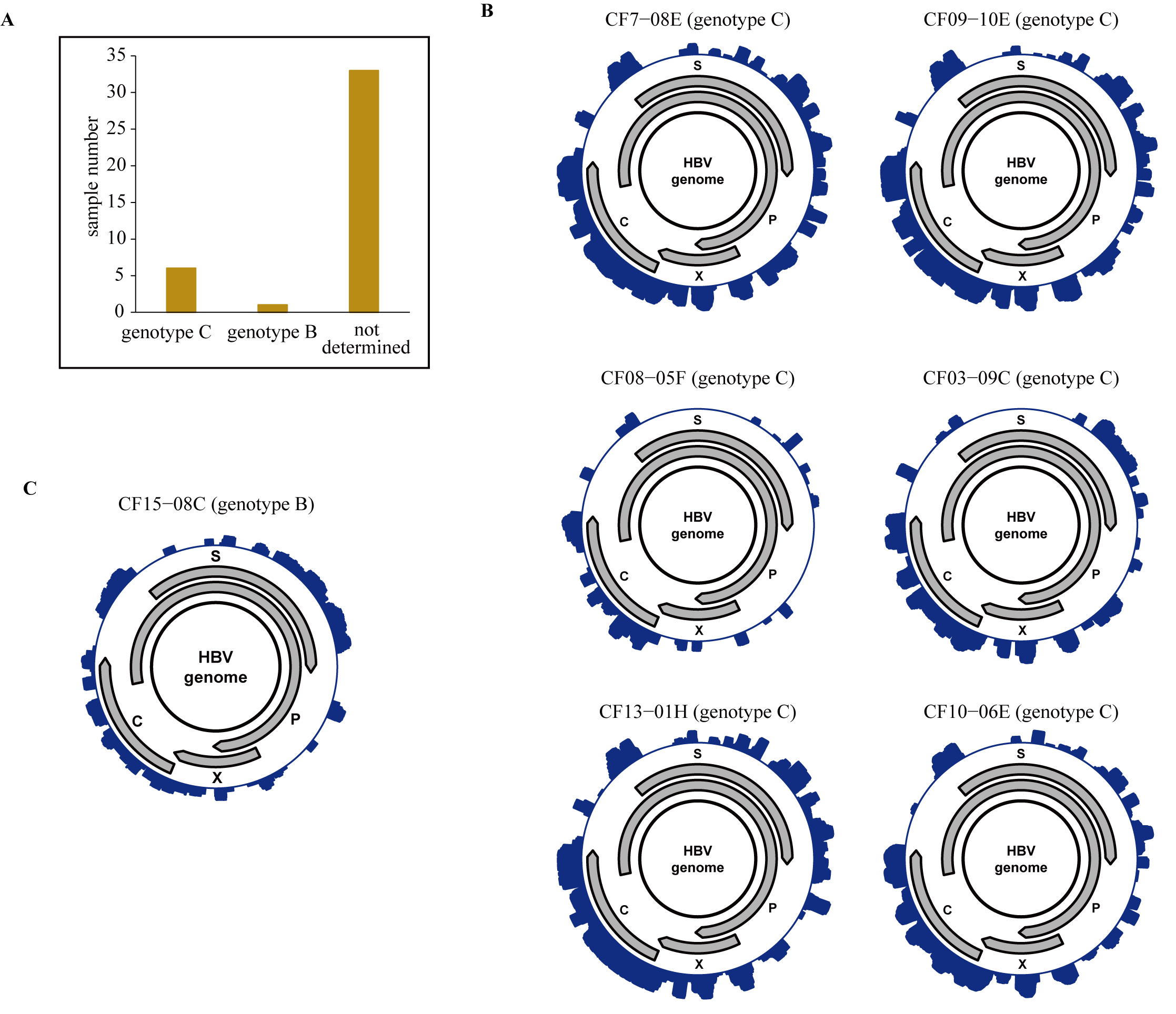

Supplement: Supplementary file 3 — Supplementary Figure 3. The genotype and reads coverage of HBV RNA. (A) HBV genotypes determined by cfRNA in HBV‐infected patients. (B‐C) The distribution of cfRNA reads mapping to HBV genotype C (B) and genotype B (C) genome. [file CTM2-13-e1439-s009.tif]

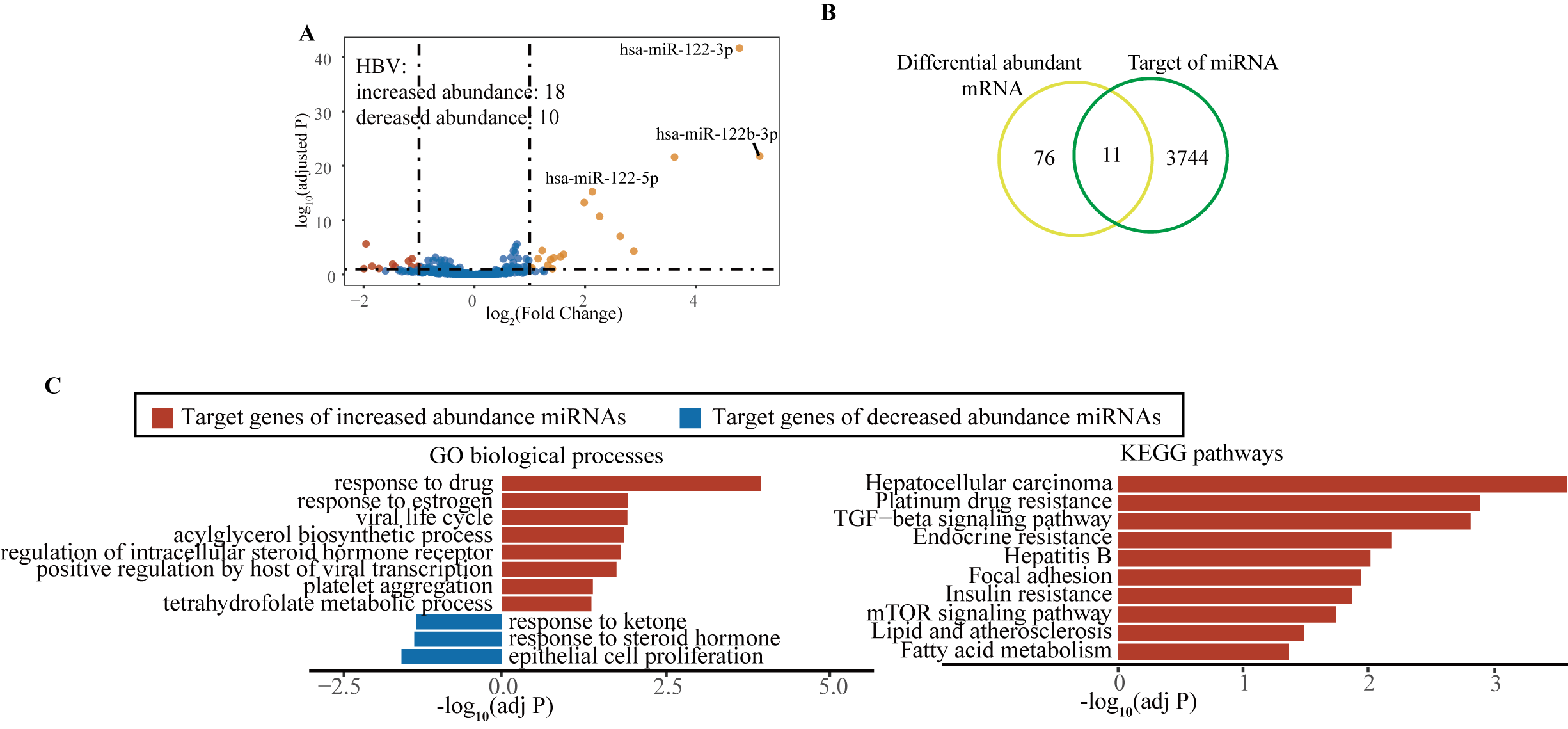

Supplement: Supplementary file 4 — Supplementary Figure 4. The target genes of differentially abundant miRNAs of the HBV groups. (A) Volcano plot of the differentially abundant cf‐miRNAs of patients with HBV infection. (B) The overlap of target genes of differentially abundant miRNAs and differentially abundant mRNAs in the HBV group. (C) The enrichment pathway of predicted target genes of differentially abundant miRNAs of HBV. [file CTM2-13-e1439-s008.tif]

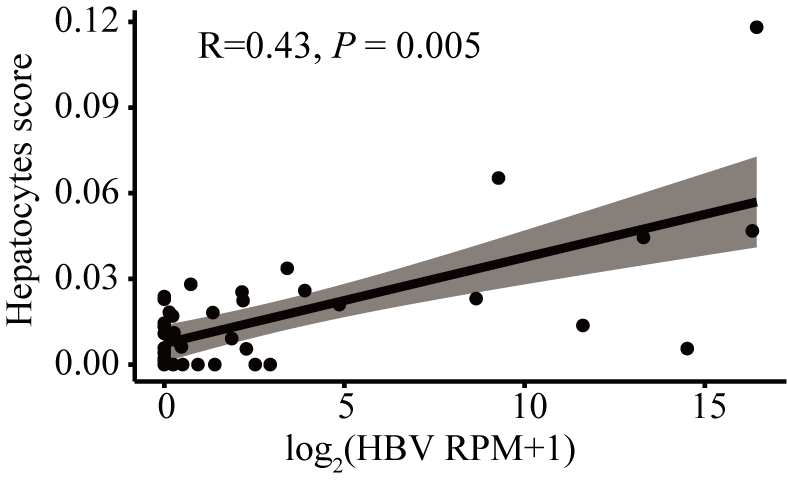

Supplement: Supplementary file 5 — Supplementary Figure 5. The Spearman correlations of hepatocytes and the HBV RNA level. [file CTM2-13-e1439-s005.tif]

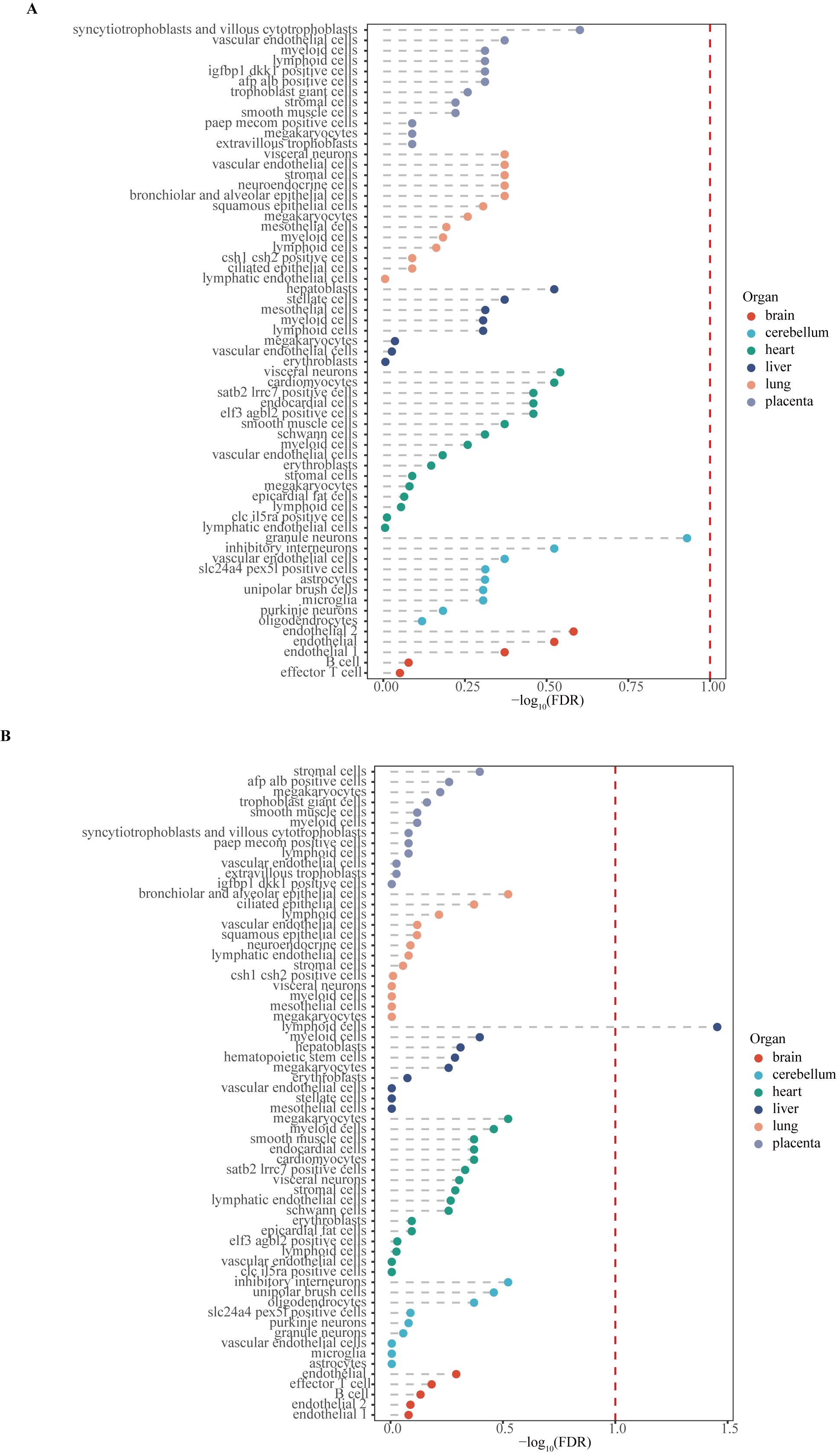

Supplement: Supplementary file 6 — Supplementary Figure 6. Fetal cell‐specific signature scores in the ICP and HBV groups. (A‐B) The cumulative signature score of the fetal liver, brain, lung and heart, and placenta in ICP (A) and HBV (B) groups. X‐axis is the false discovery rate (FDR) comparing the fetal signature score between patients and healthy pregnant women. P values were calculated using Wilcoxon's rank‐sum test. [file CTM2-13-e1439-s001.tif]

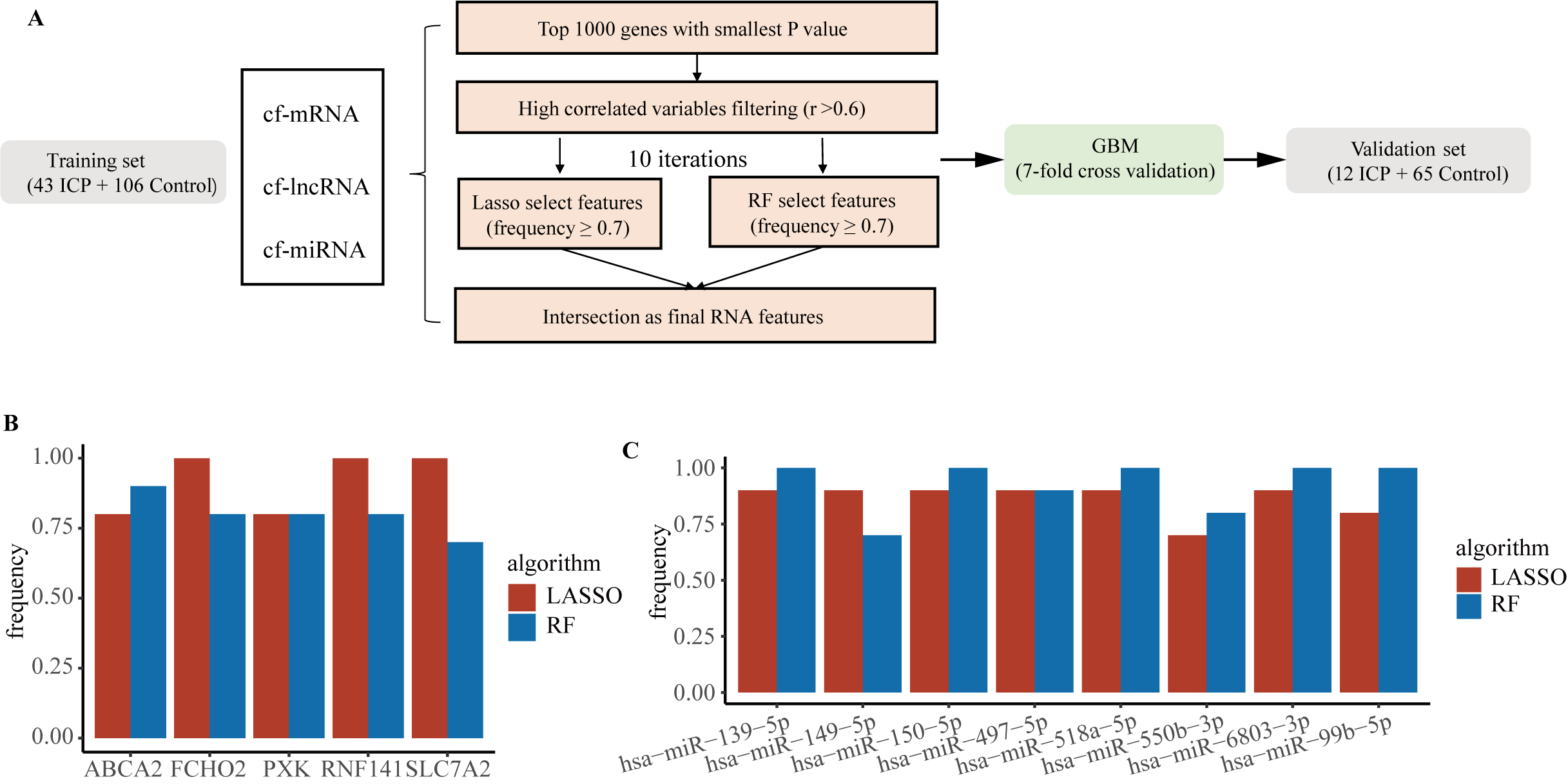

Supplement: Supplementary file 7 — Supplementary Figure 7. The feature selection process of the ICP prediction model. (A) Detailed process of ICP prediction model construction. (B‐C) The frequency of the final feature mRNA (B) and miRNA (C) genes when selected by LASSO and RF algorithms, respectively. LASSO, least absolute shrinkage and selection operator; RF, random forest; GBM, gradient boosting machine. [file CTM2-13-e1439-s007.tif]
