# Supplementary material for: Early characterisation and prediction of liver diseases in pregnancy by plasma cell‐free RNAs
Source: Clin Transl Med. 2023 Oct 13;13(10):e1439. doi: 10.1002/ctm2.1439 (PMC10570770; doi:10.1002/ctm2.1439)
Supplement: Supplementary file 8 — Supporting Information [file CTM2-13-e1439-s004.docx]

**Materials and Methods**

**Plasma samples collection**

Peripheral blood samples were collected from singleton pregnancies in the second trimester of pregnancy (ranging from 13 weeks to 25 weeks). A total of 307 subjects (HBV, N=47; ICP=89; CTL=171) were recruited for this study and all of the subjects were at least 18 years old. Women presenting with classical pruritus with no rash and raised serum TBA (> 10 μmol/L) (all of which subsequently disappeared after delivery) were diagnosed with ICP. Women who had signs of chronic liver diseases, viral hepatitis infection, and skin diseases were excluded from the ICP group. Control pregnant women were selected according to the gestational age of blood collection and fetus gender similar to women with ICP and HBV. Pregnant women subsequently diagnosed with preterm birth, preeclampsia, gestational diabetes, and placental abruption were excluded from the control group. All pregnancies resulted in live births. We collected detailed clinical information including laboratory test results for each participant, and women who missed more than 60% of clinical information or alanine aminotransferase (ALT), aspartate aminotransferase (AST) and TBA were also excluded from the analysis (ICP, N=0; HBV, N=1; CTL, N=0).

**Plasma sample processing and sequencing**

Whole blood samples were collected using EDTA-containing tubes and processed within 8 hours. Plasma was separated according to a two-step centrifugation protocol: 1,600 g for 10 min at 4℃ and 12,000 g for 10 min at 4℃. The resulting plasma supernatant was stored at -80°C until use. Total cfRNA was extracted from 250 uL plasma with 750 uL TRIZOL LS Reagent (Thermo Fisher, 10296028) according to the manufacturer’s protocol. The transcriptome library was constructed using the Polyadenylation ligation-mediated sequencing (PALM-Seq) method ^1^, which enables to obtain mRNA, lncRNA, and small RNA in a single library. cfRNA sequencing was performed on the MGISEQ-2000 platform (single-end 50 bp) with a depth of more than 32 million reads per sample.

**Cf-RNA alignment and quantification**

Adapter and low-quality reads were trimmed, then reads with more than 10% ‘N’ base or shorter than 17 bp were filtered by cutadapt ^2^. The reads with > 70% ‘A’ base were also filtered by SOAPnuke ^3^. The clean reads were first aligned to rRNA, vault RNA, and Y RNA to remove these RNAs. Then, the remaining RNA was aligned in a specific order: 1) miRNA from the miRBase database (http://www.mirbase.org/); 2) tRNA from gtRNAdb (http://gtrnadb.ucsc.edu/) and piRNA from piRBase (http://bigdata.ibp.ac.cn/piRBase/); 3) mRNA and lncRNA from the GENCODE database (https://www.gencoDBGenes.org/) and 4) the other RNA biotypes in GENCODE. The transcriptome alignment was conducted using bowtie ^4^. Gene-level expression of mRNA and lncRNA was estimated by RSEM and was normalized to transcripts per kilobase million mapped reads (TPM) ^5^. The abundance of miRNA was calculated by reads per million mapped reads (RPM). Reads that failed to map to the transcriptome were then aligned to the human genome using STAR ^6^.

Samples that had a detected mRNA number (TPM > 0) smaller than 6000 or lncRNA number (TPM > 0) smaller than 1900 or miRNA number (RPM > 0) smaller than 140 were excluded from further analysis (HBV, N=6; ICP, N=15; CTL, N=0). Finally, a total of 285 subjects (HBV, N=40; ICP=74; CTL=171) were included in this study. On average, we identified 9552 mRNAs, 2934 lncRNAs, and 470 miRNAs per sample. Forty patients carried HBV, including 10 patients with positive HBeAg and 30 patients with negative HBeAg. A total of 74 subjects were included in the ICP group, of which 19 subjects (ICP_TBA>10 group) had a high concentration of TBA (TBA ≥ 10 μmol/L) at the blood sampling age and the remaining 55 subjects (preICP group) had normal total bile acid levels (TBA < 10 μmol/L) at the time of blood collection but were subsequently diagnosed with ICP.

**HBV RNA identification and assembly**

Reads that failed to map to the human genome were finally mapped to the HBV sequences from NCBI-NT database using Kraken ^7^. The abundance of HBV was normalized to RPM. We assembled reads classified as HBV using SPAdes ^8^ and then used the top five longest contigs to determine HBV genotype in HBVdb (https://hbvdb.lyon.inserm.fr/HBVdb/).

**Differentially abundant genes (DAGs) and pathway enrichment analysis**

Genes that had no expression in ≥ 70% of the samples were excluded from DAG analysis and gene set enrichment analysis (GSEA). DAG analysis was performed using the DESeq2 package. Genes were considered as DAG if log2 fold change (FC) ≥ 1 and Benjamini-Hochberg (BH) adjusted *P* < 0.1. The pathway enrichment analysis and GSEA were performed using clusterProfile R package ^9^. A pathway with a BH-adjusted *P*-value < 0.05 was considered as a significant enrichment. The miRNA target genes were predicted with the miRTarBase database (https://mirtarbase.cuhk.edu.cn/), which is an experimentally validated miRNA-target database. In the single sample GSEA (ssGSEA), only significantly enriched GSEA pathways were included and the enrichment score was calculated for each sample with GSVA ^10^.

**Tissue- / cell-specific signature calculation**

Tissue-specific mRNA genes were downloaded from The Human Protein Atlas database (HPA, version 21). To ensure the specificity of the gene’s tissue origin, we only included tissue-enriched genes, which has at least four-fold higher mRNA level in a particular tissue compared to any other tissue. Male-specific tissues (such as the testis and prostate) were not considered in this study. A miRNA gene was considered tissue-specific with a tissue specificity index ≥ 0.8 in the TissueAtlas database (https://www.ccb.uni-saarland.de/tissueatlas2). We focused on the placenta and fetal heart, lungs, and brain tissues, as these may be affected by elevated levels of bile acids ^11^. The cell-specific genes of the fetal liver, brain, lung, heart, and placenta were obtained from Molecular Signatures Database C8 (https://www.gsea-msigdb.org/gsea/msigdb/). Liver- and fetal-specific signature scores were calculated by cumulating the expression level of their specific gene sets ^12^. Signature score for hepatocyte was calculated using xCell ^13^. Correlations between hepatocyte-specific signature and liver function indicators were calculated with Spearman correlation coefficients and *P* < 0.05 was considered statistically significant.

**ICP predictive model construction**

The predictive model was constructed using plasma cf-mRNA, cf-lncRNA and cf-miRNA, respectively. Samples from the preICP group were split into training (collected from February 2017 to December 2018) and validation (Collected from January 2016 to January 2017) sets according to the time of blood collection. Healthy controls in the training set were selected based on the gestational week of blood collection, age, and fetal gender of ICP samples, and the remaining samples were used as the validation set. The feature selection included two steps. In step 1, we first conducted differentially abundant gene analyses in the training set, then selected 1000 genes (all of the miRNA genes were selected) with the smallest *P*-value as candidate features to remove excessive noise and reduce training time cost. In step 2, we removed highly correlated variables (Pearson correlation coefficient > 0.6) and perform log2 (TPM+1) transformation on the candidate features and center and scaled it to zero mean and one standard deviation. We then used the least absolute shrinkage and selection operator (LASSO) and random forest (RF) with multiple-split iterations (10 iterations) to select features, and genes with frequency ≥ 0.7 were selected as RF features and LASSO features respectively. The intersection of these two types of features was finally selected to construct models. RF and LASSO were built upon the randomForest and glmnet packages in R. Gradient boost machine (GBM) was used for model construction with gbm package and used 7-fold cross-validation to select the best hyperparameter. The receiver operating characteristic curve (AUROC) is an evaluation index. After the hyperparameter selection, all training data will be used to refit the model and evaluated the model performance in the validation set. All training workflow was performed in R software with caret packages. AUROC calculation used the pROC packages and the confusion matrix calculation used the caret package. The best cut-off risk probability was determined using the Youden index.

**Statistical analysis**

Statistical analysis between groups was performed using Wilcoxon’s rank-sum test for continuous variables and Fisher’s exact test for categorical variables. Statistical analyses and data visualization were performed with R software (version 3.5.1). All tests were two-tailed and *P* < 0.05 was considered statistically significant. For multiple testing, a BH-corrected *P*-value < 0.1 was considered significant.

**Reference**

1. Liu Z, Wang T, Yang X, et al. Polyadenylation ligation-mediated sequencing (PALM-Seq) characterizes cell-free coding and non-coding RNAs in human biofluids. *Clin Transl Med*. Jul 2022;12(7):e987. doi:10.1002/ctm2.987

2. Martin M. Cutadapt removes adapter sequences from high-throughput sequencing reads. *EMBnet journal*. Nov 2011;17(1):10-12. doi:10.1089/cmb.2017.0096

3. Chen Y, Chen Y, Shi C, et al. SOAPnuke: a MapReduce acceleration-supported software for integrated quality control and preprocessing of high-throughput sequencing data. *Gigascience*. Jan 1 2018;7(1):gix120. doi:10.1093/gigascience/gix120

4. Langmead B, Trapnell C, Pop M, Salzberg SL. Ultrafast and memory-efficient alignment of short DNA sequences to the human genome. *Genome Biol*. 2009;10(3):R25. doi:10.1186/gb-2009-10-3-r25

5. Li B, Dewey CN. RSEM: accurate transcript quantification from RNA-Seq data with or without a reference genome. *BMC Bioinformatics*. Aug 4 2011;12:323. doi:10.1186/1471-2105-12-323

6. Dobin A, Davis CA, Schlesinger F, et al. STAR: ultrafast universal RNA-seq aligner. *Bioinformatics*. Jan 1 2013;29(1):15-21. doi:10.1093/bioinformatics/bts635

7. Wood DE, Salzberg SL. Kraken: ultrafast metagenomic sequence classification using exact alignments. *Genome Biol*. Mar 3 2014;15(3):R46. doi:10.1186/gb-2014-15-3-r46

8. Bankevich A, Nurk S, Antipov D, et al. SPAdes: a new genome assembly algorithm and its applications to single-cell sequencing. *J Comput Biol*. May 2012;19(5):455-77. doi:10.1089/cmb.2012.0021

9. Yu G, Wang LG, Han Y, He QY. clusterProfiler: an R package for comparing biological themes among gene clusters. *OMICS*. May 2012;16(5):284-7. doi:10.1089/omi.2011.0118

10. Hanzelmann S, Castelo R, Guinney J. GSVA: gene set variation analysis for microarray and RNA-seq data. *BMC Bioinformatics*. Jan 16 2013;14:7. doi:10.1186/1471-2105-14-7

11. Zhang L, Zhang F, Tang L, Yang W, Zhang X. Intrahepatic cholestasis of pregnancy and fetal injury. *Zhong Nan Da Xue Xue Bao Yi Xue Ban*. Jun 2013;38(6):645-52. doi:10.3969/j.issn.1672-7347.2013.06.015

12. Rasmussen M, Reddy M, Nolan R, et al. RNA profiles reveal signatures of future health and disease in pregnancy. *Nature*. Jan 2022;601(7893):422-427. doi:10.1038/s41586-021-04249-w

13. Aran D, Hu Z, Butte AJ. xCell: digitally portraying the tissue cellular heterogeneity landscape. *Genome Biol*. Nov 15 2017;18(1):220. doi:10.1186/s13059-017-1349-1
