# Supplementary material for: Early characterisation and prediction of liver diseases in pregnancy by plasma cell‐free RNAs
Source: Clin Transl Med. 2023 Oct 13;13(10):e1439. doi: 10.1002/ctm2.1439 (PMC10570770; doi:10.1002/ctm2.1439)
Supplement: Supplementary file 9 — Supporting Information [file CTM2-13-e1439-s006.docx]

**Supplementary tables**

| **Supplementary Table 1. Clinical characteristics of subjects with ICP.** | | | | | |  |  |
| --- | --- | --- | --- | --- | --- | --- | --- |
| **Characteristics** | **CTL (N=171)** | **ICP (N=74)** | **preICP (N=55)** | **ICP_TBA10 (N=19)** | ***P* value (ICP *vs.* CTL)** | ***P* value (preICP *vs.* CTL)** | ***P* value (ICP_TBA10 *vs.* CTL)** |
| Sampling GA (week) | 16.98(±2.52) | 17.28(±2.86) | 17.32 (±2.85) | 17.19(±2.96) | 0.461 | 0.444 | 0.819 |
| Diagnosis GA (week) | n/a | 27.47(±11.07) | 33.20 (±5.82) | 10.89 (±2.28) | n/a | n/a | n/a |
| Delivery GA (week) | 39.9(±0.68) | 37.35(±2.16) | 37.31 (±2.33) | 37.49(±1.6) | 4.632E-24 | 1.403E-19 | 4.211E-09 |
| Male fetus (%) | 108(63.16) | 38(51.35) | 31 (56.36) | 7(36.84) | 0.091 | 0.426 | 0.045 |
| Maternal age (year) | 32.66(±3.61) | 32.66(±4.19) | 32.38 (±3.97) | 33.47(±4.8) | 0.917 | 0.609 | 0.476 |
| Pre-pregnancy BMI (kg/m2) | 20.74(±2.21) | 20.57(±2.74) | 20.32 (±2.69) | 21.31(±2.83) | 0.411 | 0.145 | 0.390 |
| Preterm birth | 0(0) | 25(33.78) | 18 (32.73) | 7(36.84) | 3.604E-15 | 7.815E-13 | 3.177E-08 |
| Onset of labor, n (%) |  |  |  |  |  |  |  |
| Spontaneous | 96 (56.14) | 16 (21.62) | 14 (25.45) | 2 (10.53) |  |  |  |
| Cesarean section | 75 (43.86) | 58 (77.33) | 41 (75.55) | 17 (89.47) | 0.000 | 0.000 | 0.000 |
| TBA (µmol/L) | 1.77(±1.06) | 6.65(±6.93) | 3.25 (±2.5) | 16.5(±6.19) | 6.712E-12 | 9.201E-06 | 8.923E-13 |
| ALT (U/L) | 13.8(±6.17) | 21.05(±17.45) | 22.53 (±18.73) | 16.79(±12.51) | 3.523E-04 | 3.481E-05 | 0.746 |
| AST (U/L) | 17.92(±3.58) | 21.09(±8.24) | 21.56 (±8.94) | 19.74(±5.75) | 0.005 | 0.005 | 0.305 |
| GGT (U/L) | 13.43(±6.15) | 17.04(±11.13) | 18.45 (±12.09) | 12.95(±6.31) | 0.011 | 0.001 | 0.851 |
| TP (g/L) | 71.17(±3.5) | 71.5(±3.39) | 71.75 (±3.51) | 70.77(±3.02) | 0.554 | 0.275 | 0.491 |
| Albumin (g/L) | 41.94(±2.29) | 42.15(±2.07) | 42.38 (±1.96) | 41.49(±2.28) | 0.477 | 0.225 | 0.521 |
| Globulin (g/L) | 29.24(±3.06) | 29.35(±2.98) | 29.38 (±3.05) | 29.27(±2.84) | 0.693 | 0.706 | 0.856 |
| Albumin /Globulin | 1.45(±0.19) | 1.45(±0.19) | 1.46 (±0.18) | 1.43(±0.21) | 0.795 | 0.903 | 0.422 |
| BIL-T (µmol/L) | 7.71(±3.28) | 8.15(±2.87) | 8.11 (±3.09) | 8.25(±2.19) | 0.108 | 0.283 | 0.108 |
| BIL-D (µmol/L) | 3.26(±1.41) | 3.38(±1.22) | 3.35 (±1.32) | 3.49(±0.9) | 0.289 | 0.610 | 0.154 |
| Triglyceride (mmol/L) | 1.35(±0.59) | 1.34(±0.46) | 1.32 (±0.45) | 1.41(±0.48) | 0.485 | 0.756 | 0.328 |
| ALP (U/L) | 53.18(±32.16) | 52.48(±34.52) | 51.43 (±34.34) | 55.47(±35.81) | 0.700 | 0.864 | 0.590 |
| Cholesterol (mmol/L) | 4.62(±0.76) | 4.37(±0.78) | 4.43 (±0.79) | 4.2(±0.75) | 0.010 | 0.064 | 0.021 |
| HDL (mmol/L) | 1.92(±0.37) | 1.89(±0.36) | 1.94 (±0.36) | 1.76(±0.36) | 0.863 | 0.503 | 0.107 |
| LDL (mmol/L) | 2.64(±0.67) | 2.42(±0.69) | 2.45 (±0.7) | 2.33(±0.67) | 0.008 | 0.024 | 0.080 |

Continuous variables were presented as mean ± SD. Categorical variables were presented as the frequency and proportion of patients in each category. *P* values were calculated using Fisher’s exact test for categorical variables and using Wilcoxon’s rank-sum test for continuous variables. GA, gestational age; BMI, body mass index; TBA, total biliary acid; ALT, alanine aminotransferase; AST, aspartate aminotransferase; GGT, γ-glutamyl transpeptidase; TP, total protein; BIL-T, total bilirubin; BIL-D, direct bilirubin; ALP, alkaline phosphatase; HDL, high-density lipoprotein; LDL, low-density lipoprotein. n/a, not applicable.

| **Supplementary Table 2. Different abundance mRNA, miRNA and lncRNA.** | | | | | |  |
| --- | --- | --- | --- | --- | --- | --- |
| **GeneID** | **log_2_FC(HBV/CTL) or log_2_FC(ICP/CTL)** | ***P*adj** | ***P* value** | **RNA type** | **diseases** | **tissue** |
| ABCA2 | 1.52 | 0.086 | 0.000 | mRNA | ICP | brain |
| ABCB11 | 1.87 | 0.065 | 0.000 | mRNA | HBV | liver |
| ABCB4 | 2.52 | 0.073 | 0.001 | mRNA | HBV | liver |
| AC004156.1 | -2.40 | 0.000 | 0.000 | lncRNA | HBV | NA |
| AC024614.4 | 1.51 | 0.054 | 0.000 | lncRNA | HBV | NA |
| AC026412.3 | -2.08 | 0.052 | 0.000 | lncRNA | HBV | NA |
| AIPL1 | 1.88 | 0.097 | 0.001 | mRNA | HBV | retina |
| AL353583.1 | 1.99 | 0.000 | 0.000 | mRNA | ICP | NA |
| AL451123.1 | 2.12 | 0.021 | 0.000 | lncRNA | HBV | NA |
| AL591885.1 | 2.31 | 0.047 | 0.000 | lncRNA | HBV | NA |
| ALB | 2.03 | 0.000 | 0.000 | mRNA | HBV | liver |
| ALDH8A1 | 1.38 | 0.023 | 0.000 | mRNA | HBV | NA |
| ALDOB | 1.97 | 0.000 | 0.000 | mRNA | HBV | NA |
| AP3B2 | 1.55 | 0.025 | 0.000 | mRNA | HBV | NA |
| APOH | 1.86 | 0.000 | 0.000 | mRNA | HBV | liver |
| ASL | 1.69 | 0.093 | 0.000 | mRNA | ICP | liver |
| ASPH | 1.07 | 0.019 | 0.000 | mRNA | HBV | NA |
| C1S | 1.21 | 0.090 | 0.001 | mRNA | HBV | liver |
| C2orf88 | -1.11 | 0.002 | 0.000 | mRNA | HBV | NA |
| C7orf26 | -2.08 | 0.090 | 0.001 | mRNA | HBV | NA |
| CACYBP | 1.75 | 0.037 | 0.000 | mRNA | HBV | NA |
| CAMP | -2.09 | 0.067 | 0.001 | mRNA | HBV | bone marrow |
| CARS | 1.50 | 0.033 | 0.000 | mRNA | HBV | NA |
| CDC34 | 1.53 | 0.008 | 0.000 | mRNA | HBV | NA |
| CDKN1C | 1.45 | 0.008 | 0.000 | mRNA | HBV | NA |
| CEP70 | 1.28 | 0.033 | 0.000 | mRNA | HBV | NA |
| CFH | 3.58 | 0.000 | 0.000 | mRNA | HBV | liver |
| CHST14 | -2.28 | 0.083 | 0.001 | mRNA | HBV | NA |
| CP | 1.85 | 0.095 | 0.001 | mRNA | HBV | liver |
| CPB2 | 2.84 | 0.010 | 0.000 | mRNA | HBV | liver |
| CSRNP1 | 1.59 | 0.003 | 0.000 | mRNA | HBV | NA |
| CYP2E1 | 2.08 | 0.000 | 0.000 | mRNA | HBV | liver |
| DBN1 | 1.00 | 0.048 | 0.000 | mRNA | ICP | NA |
| DCTN3 | 1.61 | 0.012 | 0.000 | mRNA | ICP | NA |
| DCTN3 | 1.62 | 0.070 | 0.001 | mRNA | HBV | NA |
| DPP9 | 1.32 | 0.000 | 0.000 | mRNA | HBV | NA |
| DTX1 | -2.17 | 0.033 | 0.000 | mRNA | HBV | NA |
| ECE1 | -1.57 | 0.033 | 0.000 | mRNA | HBV | NA |
| EPB41L5 | 2.71 | 0.000 | 0.000 | mRNA | HBV | NA |
| ERCC2 | -2.06 | 0.065 | 0.000 | mRNA | HBV | NA |
| F9 | 3.03 | 0.013 | 0.000 | mRNA | HBV | liver |
| FAM13A | 1.78 | 0.037 | 0.000 | mRNA | HBV | NA |
| FAM71D | 1.13 | 0.089 | 0.000 | mRNA | ICP | NA |
| FAM72A | 2.08 | 0.073 | 0.001 | mRNA | HBV | NA |
| FAM83H | 1.22 | 0.097 | 0.000 | mRNA | ICP | NA |
| FASN | 1.39 | 0.067 | 0.001 | mRNA | HBV | NA |
| FBXO45 | 1.21 | 0.053 | 0.000 | mRNA | HBV | NA |
| FGA | 1.52 | 0.019 | 0.000 | mRNA | HBV | liver |
| FGB | 1.60 | 0.040 | 0.000 | mRNA | HBV | liver |
| FGG | 1.64 | 0.065 | 0.000 | mRNA | HBV | liver |
| FKBP3 | -1.16 | 0.050 | 0.000 | mRNA | HBV | NA |
| FKTN | -1.87 | 0.047 | 0.000 | mRNA | ICP | NA |
| GHR | 1.38 | 0.039 | 0.000 | mRNA | HBV | NA |
| GRIN2B | 2.33 | 0.000 | 0.000 | mRNA | HBV | brain |
| GRM4 | 1.92 | 0.096 | 0.000 | mRNA | ICP | brain |
| HHAT | 1.01 | 0.011 | 0.000 | mRNA | ICP | NA |
| HOXC10 | -1.10 | 0.036 | 0.000 | mRNA | HBV | skeletal muscle |
| HRG | 3.84 | 0.000 | 0.000 | mRNA | HBV | liver |
| HRNR | 1.80 | 0.036 | 0.000 | mRNA | HBV | NA |
| hsa-miR-122-3p | 4.79 | 0.000 | 0.000 | miRNA | HBV | liver |
| hsa-miR-122-5p | 2.13 | 0.000 | 0.000 | miRNA | HBV | liver |
| hsa-miR-122b-3p | 5.15 | 0.000 | 0.000 | miRNA | HBV | NA |
| hsa-miR-181a-3p | -1.13 | 0.001 | 0.000 | miRNA | HBV | NA |
| hsa-miR-192-3p | 1.33 | 0.019 | 0.001 | miRNA | HBV | liver |
| hsa-miR-192-5p | 1.98 | 0.000 | 0.000 | miRNA | HBV | liver |
| hsa-miR-194-5p | 2.26 | 0.000 | 0.000 | miRNA | HBV | liver |
| hsa-miR-1972 | 1.24 | 0.038 | 0.000 | miRNA | ICP | NA |
| hsa-miR-25-5p | -1.85 | 0.031 | 0.002 | miRNA | HBV | NA |
| hsa-miR-30c-5p | 1.03 | 0.000 | 0.000 | miRNA | ICP | NA |
| hsa-miR-3200-5p | -1.48 | 0.013 | 0.001 | miRNA | HBV | brain |
| hsa-miR-374c-3p | 1.41 | 0.087 | 0.011 | miRNA | HBV | NA |
| hsa-miR-378a-3p | 1.22 | 0.000 | 0.000 | miRNA | HBV | muscle |
| hsa-miR-378d | 1.15 | 0.001 | 0.000 | miRNA | HBV | muscle |
| hsa-miR-378g | 1.61 | 0.000 | 0.000 | miRNA | HBV | NA |
| hsa-miR-378h | 1.39 | 0.022 | 0.000 | miRNA | ICP | muscle |
| hsa-miR-378i | 1.38 | 0.002 | 0.000 | miRNA | HBV | muscle |
| hsa-miR-4433b-5p | -1.96 | 0.000 | 0.000 | miRNA | HBV | testis |
| hsa-miR-4443 | 2.88 | 0.000 | 0.000 | miRNA | HBV | NA |
| hsa-miR-4443 | 1.85 | 0.009 | 0.000 | miRNA | ICP | NA |
| hsa-miR-4454 | 1.55 | 0.001 | 0.000 | miRNA | HBV | NA |
| hsa-miR-455-3p | 3.62 | 0.000 | 0.000 | miRNA | HBV | NA |
| hsa-miR-455-5p | 2.63 | 0.000 | 0.000 | miRNA | HBV | NA |
| hsa-miR-4800-3p | 1.43 | 0.079 | 0.002 | miRNA | ICP | NA |
| hsa-miR-499a-5p | 1.02 | 0.087 | 0.011 | miRNA | HBV | muscle |
| hsa-miR-501-5p | -1.43 | 0.034 | 0.003 | miRNA | HBV | NA |
| hsa-miR-5585-3p | -2.00 | 0.084 | 0.010 | miRNA | HBV | NA |
| hsa-miR-574-5p | 1.43 | 0.001 | 0.000 | miRNA | HBV | NA |
| hsa-miR-625-5p | -1.02 | 0.033 | 0.003 | miRNA | HBV | NA |
| hsa-miR-6803-3p | -1.13 | 0.037 | 0.003 | miRNA | HBV | NA |
| hsa-miR-8058 | -1.72 | 0.078 | 0.009 | miRNA | HBV | NA |
| hsa-miR-874-3p | -1.19 | 0.003 | 0.000 | miRNA | HBV | NA |
| hsa-miR-92a-1-5p | 1.04 | 0.063 | 0.007 | miRNA | HBV | NA |
| hsa-miR-9901 | 1.40 | 0.048 | 0.001 | miRNA | ICP | bowel |
| IBTK | 1.19 | 0.000 | 0.000 | mRNA | HBV | NA |
| IFIT1 | 1.19 | 0.037 | 0.000 | mRNA | HBV | NA |
| IGF1 | 1.38 | 0.068 | 0.001 | mRNA | HBV | NA |
| INHBA | 1.13 | 0.000 | 0.000 | mRNA | ICP | NA |
| IQSEC2 | 2.24 | 0.000 | 0.000 | mRNA | ICP | NA |
| ITPR2 | 1.08 | 0.017 | 0.000 | mRNA | HBV | NA |
| KIAA1671 | 1.58 | 0.061 | 0.000 | mRNA | ICP | NA |
| KLHL36 | -2.23 | 0.069 | 0.001 | mRNA | HBV | NA |
| LINC00477 | 2.02 | 0.021 | 0.000 | lncRNA | HBV | NA |
| LINC01629 | -2.84 | 0.011 | 0.000 | lncRNA | HBV | NA |
| LPAR5 | -2.25 | 0.083 | 0.001 | mRNA | HBV | NA |
| LRP6 | 1.47 | 0.033 | 0.000 | mRNA | HBV | NA |
| LXN | 2.29 | 0.069 | 0.001 | mRNA | HBV | NA |
| MAN1A2 | 1.07 | 0.002 | 0.000 | mRNA | HBV | NA |
| MEF2C-AS1 | -1.75 | 0.091 | 0.000 | lncRNA | HBV | NA |
| MIA2 | 1.52 | 0.000 | 0.000 | mRNA | HBV | NA |
| MIA3 | 1.27 | 0.069 | 0.001 | mRNA | HBV | NA |
| MTHFD2L | 2.07 | 0.022 | 0.000 | mRNA | HBV | NA |
| MTRNR2L1 | -1.12 | 0.002 | 0.000 | mRNA | HBV | NA |
| MTRNR2L10 | -1.37 | 0.001 | 0.000 | mRNA | HBV | brain |
| MTRNR2L11 | -1.49 | 0.058 | 0.000 | mRNA | HBV | brain |
| MTRNR2L12 | -1.25 | 0.001 | 0.000 | mRNA | HBV | NA |
| MTRNR2L3 | -1.41 | 0.061 | 0.000 | mRNA | HBV | NA |
| MTRNR2L8 | -1.48 | 0.026 | 0.000 | mRNA | HBV | NA |
| NIPAL3 | 1.03 | 0.002 | 0.000 | mRNA | ICP | NA |
| NR2F2-AS1 | 1.11 | 0.023 | 0.000 | lncRNA | HBV | NA |
| NUDCD1 | 2.49 | 0.000 | 0.000 | mRNA | ICP | NA |
| PDHB | 1.20 | 0.070 | 0.001 | mRNA | HBV | NA |
| PKD1P6-NPIPP1 | 1.70 | 0.014 | 0.000 | lncRNA | HBV | NA |
| PLEKHG4B | 2.18 | 0.012 | 0.000 | mRNA | ICP | NA |
| PNMA8C | -2.61 | 0.016 | 0.000 | mRNA | HBV | NA |
| POLR3A | 1.21 | 0.011 | 0.000 | mRNA | HBV | NA |
| PROSER3 | 1.32 | 0.000 | 0.000 | mRNA | ICP | NA |
| PROSER3 | 1.18 | 0.003 | 0.000 | mRNA | HBV | NA |
| PTER | -1.61 | 0.048 | 0.000 | mRNA | ICP | NA |
| RGL3 | 2.73 | 0.037 | 0.000 | mRNA | HBV | NA |
| RGS12 | 1.05 | 0.015 | 0.000 | mRNA | ICP | NA |
| SCD | 1.40 | 0.047 | 0.000 | mRNA | ICP | NA |
| SCD | 1.54 | 0.034 | 0.000 | mRNA | HBV | NA |
| SEPT10 | 1.10 | 0.027 | 0.000 | mRNA | ICP | NA |
| SERPINH1 | 1.30 | 0.012 | 0.000 | mRNA | ICP | NA |
| SESTD1 | -1.26 | 0.020 | 0.000 | mRNA | HBV | NA |
| SHF | 1.39 | 0.079 | 0.000 | mRNA | ICP | NA |
| SKOR2 | 1.67 | 0.033 | 0.000 | mRNA | HBV | NA |
| SLC2A2 | 2.89 | 0.011 | 0.000 | mRNA | HBV | liver |
| SLC2A4 | 1.28 | 0.036 | 0.000 | mRNA | HBV | NA |
| SLC38A4 | 2.74 | 0.000 | 0.000 | mRNA | HBV | liver |
| SLC41A2 | 2.18 | 0.001 | 0.000 | mRNA | HBV | NA |
| SLC41A3 | 1.92 | 0.008 | 0.000 | mRNA | ICP | NA |
| SLC6A18 | -2.56 | 0.069 | 0.001 | mRNA | HBV | kidney |
| SLC7A2 | 1.01 | 0.012 | 0.000 | mRNA | ICP | NA |
| SLC7A2 | 1.11 | 0.036 | 0.000 | mRNA | HBV | NA |
| SMARCAL1 | 3.86 | 0.000 | 0.000 | mRNA | HBV | NA |
| SOX5 | 2.20 | 0.069 | 0.001 | mRNA | HBV | NA |
| TENT4A | 1.20 | 0.027 | 0.000 | mRNA | ICP | NA |
| TESK2 | 1.41 | 0.068 | 0.001 | mRNA | HBV | NA |
| TMEM43 | 1.40 | 0.089 | 0.000 | mRNA | ICP | NA |
| TMEM80 | 3.76 | 0.000 | 0.000 | mRNA | HBV | NA |
| UGT2B10 | 3.82 | 0.000 | 0.000 | mRNA | HBV | liver |
| UGT2B15 | 2.93 | 0.004 | 0.000 | mRNA | HBV | NA |
| UGT2B4 | 1.73 | 0.033 | 0.000 | mRNA | HBV | liver |
| UGT2B7 | 4.66 | 0.000 | 0.000 | mRNA | HBV | NA |
| UHRF2 | 1.06 | 0.019 | 0.000 | mRNA | HBV | NA |
| XBP1 | 1.10 | 0.045 | 0.000 | mRNA | HBV | NA |
| ZNF44 | 1.23 | 0.068 | 0.001 | mRNA | HBV | NA |
| ZNF517 | 1.57 | 0.096 | 0.000 | mRNA | ICP | NA |

| **Supplementary Table 3. Clinical characteristics of subjects with HBV infection.** | | | | | | |  |
| --- | --- | --- | --- | --- | --- | --- | --- |
| **Characteristics** | **CTL (N=171)** | **HBV  (N=40)** | **HBeAg- (N=30)** | **HBeAg+ (N=10)** | ***P* value  (HBV *vs.* CTL)** | ***P* value  (HBeAg+ *vs.* CTL)** | ***P* value  (HBeAg- *vs.* CTL)** |
| Sampling GA (week) | 16.98(±2.52) | 16.86(±2.27) | 16.92(±2.13) | 16.67(±2.74) | 0.935 | 0.439 | 0.745 |
| Delivery GA (week) | 39.9(±0.68) | 36.63(±2.84) | 36.68(±3.15) | 36.49(±1.73) | 1.492E-13 | 3.878E-07 | 2.535E-09 |
| Male fetus (%) | 108(63.16) | 20(50) | 12(40) | 8(80) | 0.151 | 0.335 | 0.025 |
| Maternal age (year) | 32.66(±3.61) | 35.1(±3.3) | 35.27(±3.27) | 34.6(±3.53) | 8.018E-05 | 0.081 | 2.097E-04 |
| Pre-pregnancy BMI (kg/m2) | 20.74(±2.21) | 21.4(±3.19) | 21.37(±3.55) | 21.47(±1.83) | 0.236 | 0.139 | 0.555 |
| TBA (µmol/L) | 1.77(±1.06) | 3.33(±3.43) | 2.67(±2.49) | 5.28(±5.04) | 8.104E-05 | 0.001 | 0.004 |
| ALT (U/L) | 13.8(±6.17) | 19.58(±14.44) | 17.87(±14.73) | 24.7(±12.85) | 0.002 | 5.740E-04 | 0.074 |
| AST (U/L) | 17.92(±3.58) | 19.62(±4.17) | 18.77(±3.93) | 22.2(±3.97) | 0.018 | 0.001 | 0.310 |
| GGT (U/L) | 13.43(±6.15) | 13.28(±5.62) | 13.3(±5.87) | 13.2(±5.07) | 1.000 | 0.801 | 0.891 |
| TP (g/L) | 71.17(±3.5) | 70.17(±3.92) | 70.87(±3.12) | 68.07(±5.36) | 0.177 | 0.044 | 0.625 |
| Albumin (g/L) | 41.94(±2.29) | 40.31(±2.74) | 40.6(±2.63) | 39.44(±3.02) | 2.455E-04 | 0.002 | 0.008 |
| Globulin (g/L) | 29.24(±3.06) | 29.86(±3.56) | 30.27(±3.39) | 28.63(±3.97) | 0.519 | 0.465 | 0.244 |
| Albumin/Globulin | 1.45(±0.19) | 1.37(±0.2) | 1.36(±0.2) | 1.4(±0.21) | 0.027 | 0.457 | 0.027 |
| BIL-T (µmol/L) | 7.71(±3.28) | 7.95(±2.62) | 7.83(±2.09) | 8.32(±3.94) | 0.238 | 0.797 | 0.210 |
| BIL-D (µmol/L) | 3.26(±1.41) | 3.23(±1) | 3.22(±0.76) | 3.26(±1.57) | 0.590 | 0.874 | 0.468 |
| Triglyceride (mmol/L) | 1.35(±0.59) | 1.5(±0.58) | 1.41(±0.57) | 1.76(±0.56) | 0.085 | 0.013 | 0.496 |
| ALP (U/L) | 53.18(±32.16) | 54.17(±22.5) | 48.23(±9.77) | 72(±37.63) | 0.008 | 0.009 | 0.094 |
| Cholesterol (mmol/L) | 4.62(±0.76) | 4.56(±0.92) | 4.39(±0.89) | 5.08(±0.84) | 0.757 | 0.089 | 0.194 |
| HDL (mmol/L) | 1.92(±0.37) | 1.92(±0.38) | 1.84(±0.35) | 2.17(±0.37) | 0.881 | 0.037 | 0.335 |
| LDL (mmol/L) | 2.64(±0.67) | 2.56(±0.72) | 2.48(±0.73) | 2.79(±0.65) | 0.583 | 0.397 | 0.264 |

Continuous variables were presented as mean ± SD. Categorical variables were presented as the frequency and proportion of patients in each category. *P* values were calculated using Fisher’s exact test for categorical variables and using Wilcoxon’s rank-sum test for continuous variables. GA, gestational age; BMI, body mass index; TBA, total biliary acid; ALT, alanine aminotransferase; AST, aspartate aminotransferase; GGT, γ-glutamyl transpeptidase; TP, total protein; BIL-T, total bilirubin; BIL-D, direct bilirubin; ALP, alkaline phosphatase; HDL, high-density lipoprotein; LDL, low-density lipoprotein.

| **Supplementary Table 4. The performance of the ICP prediction model using cf-miRNA.** | | |
| --- | --- | --- |
| Dataset | Training set | Validation set |
| True negative | 87 | 47 |
| False positive | 19 | 18 |
| False negative | 8 | 8 |
| True positive | 35 | 4 |
| Accuracy | 0.82 | 0.66 |
| Sensitivity | 0.81 | 0.33 |
| Specificity | 0.82 | 0.72 |
| AUROC | 0.9 | 0.54 |
